# Loss-of-function variant in the LRR domain of SLITRK2 implicated in a neurodevelopmental disorder

**DOI:** 10.3389/fgene.2023.1308116

**Published:** 2024-01-12

**Authors:** Tayyaba Afsar, Hongxia Fu, Hammal Khan, Zain Ali, Zamrud Zehri, Gohar Zaman, Safdar Abbas, Arif Mahmood, Qamre Alam, Junjian Hu, Suhail Razak, Muhammad Umair

**Affiliations:** ^1^ Department of Community Health Sciences, College of Applied Medical Sciences, King Saud University, Riyadh, Saudi Arabia; ^2^ King Salman Center for Disability Research, Riyadh, Saudi Arabia; ^3^ Department of Neurology, Dongguan Songshan Lake Central Hospital, Dongguan, China; ^4^ Department of Biosciences, COMSATS University Islamabad, Islamabad, Pakistan; ^5^ Department of Biochemistry, Faculty of Biological Sciences, Quaid-i-Azam University, Islamabad, Pakistan; ^6^ Department of Gynecology, Civil Hospital, Quetta, Pakistan; ^7^ Department of Computer Science, Abbottabad University of Science and Technology, Havelian, Abbottabad, Pakistan; ^8^ Department of Biological Science, Dartmouth College, Hanover, NH, United States; ^9^ Center for Medical Genetics and Hunan Key Laboratory of Medical Genetics, School of Life Sciences, Central South University, Changsha, China; ^10^ Molecular Genomics and Precision Department, ExpressMed Diagnostics and Research, Zinj, Bahrain; ^11^ Department of Central Laboratory, Dongguan Songshan Lake Central Hospital, Dongguan, China; ^12^ Medical Genomics Research Department, King Abdullah International Medical Research Center (KAIMRC), King Saud Bin Abdulaziz University for Health Sciences, Ministry of National Guard Health Affairs (MNGH), Riyadh, Saudi Arabia

**Keywords:** neurodevelopmental disorders, SLITRK2, whole-exome sequencing, novel mutation, developmental anomaly, nonsense mutation

## Abstract

**Background:** Neurodevelopmental disorders are characterized by different combinations of intellectual disability (ID), communication and social skills deficits, and delays in achieving motor or language milestones. SLITRK2 is a postsynaptic cell-adhesion molecule that promotes neurite outgrowth and excitatory synapse development.

**Methods and Results:** In the present study, we investigated a single patient segregating Neurodevelopmental disorder. SLITRK2 associated significant neuropsychological issues inherited in a rare X-linked fashion have recently been reported. Whole-exome sequencing and data analysis revealed a novel nonsense variant [c.789T>A; p.(Cys263*); NM_032539.5; NP_115928.1] in exon 5 of the *SLITRK2* gene (MIM# 300561). Three-dimensional protein modeling revealed substantial changes in the mutated SLITRK2 protein, which might lead to nonsense-medicated decay.

**Conclusion:** This study confirms the role of SLITRK2 in neuronal development and highlights the importance of including the *SLITRK2* gene in the screening of individuals presenting neurodevelopmental disorders.

## Introduction

Intellectual disability (ID) is a broad term used to describe a condition of impaired cognitive and adaptive development, also known as intellectual disability (ID) (Kriek et al., 2007). ID is classified into four subgroups based on IQ test results: (I) the mild group, with IQ scores ranging from 50 to 70; (II) the moderate group, with IQ scores ranging from 35 to 49; (III) the severe group, with IQ scores ranging from 20 to 34; and (IV) the profound group, with IQ scores below 20. The majority of individuals with ID, comprising approximately 85% of cases, fall into the mild group, while the moderate group represents the second most prevalent category, accounting for approximately 10% of cases. The severe and profound groups together make up only 5% of all ID cases (Parsamanesh and Miri-Moghaddam, 2018).

*SLITRK2* encodes the SLIT and TRK-like family member 2 protein, which belongs to the leucine-rich repeat protein family. SLITRK2 is part of a family of six vertebrate SLITRK members (SLITRK1–6) and is highly expressed in the central nervous system (Won et al., 2019). The *SLITRK2* gene shows unique expression in nascent neurons and displays the highest inhibitory impact on the outgrowth of neurites compared with other members of the *SLITRK* gene family. Its expression patterns and ability to control neurite growth are well-suited for the process of neurite development (Patapoutian and Reichardt, 2001; Aruga and Mikoshiba, 2003). *SLITRK2* has recently been linked to an X-linked intellectual developmental disorder that manifests as impaired intellectual development associated with motor, speech, and behavioral impairments. The detailed clinical description includes short stature, microcephaly/macrocephaly, kyphoscoliosis, seizures, abnormal brain MRI, thin corpus callosum, and aggressive behavior (El Chehadeh et al., 2022). *SLITRK2* is located on chromosome Xq27.3 and has five exons (8,421 bp), which encode an 845-amino-acid protein (NM_032539.5).

Patients with variants in the genes of the SLITRK family present diverse phenotypic features. For instance, *SLITRK1* variants have been associated with obsessive-compulsive and related disorders, such as Tourette’s syndrome, trichotillomania, and OCD (Abelson et al., 2005; Zuchner et al., 2006; Stein et al., 2019). Similarly, *SLITRK6* variants have been associated with neurodevelopmental disorders and linked to comorbidities of myopia and sensory deafness (El Chehadeh et al., 2022). Herein, we report a novel nonsense variant of *SLITRK2* in a boy that further confirms the role of SLITRK2 in human neurodevelopmental disorders.

## Methods

### Research subjects and study approval

**FIGURE 1:**
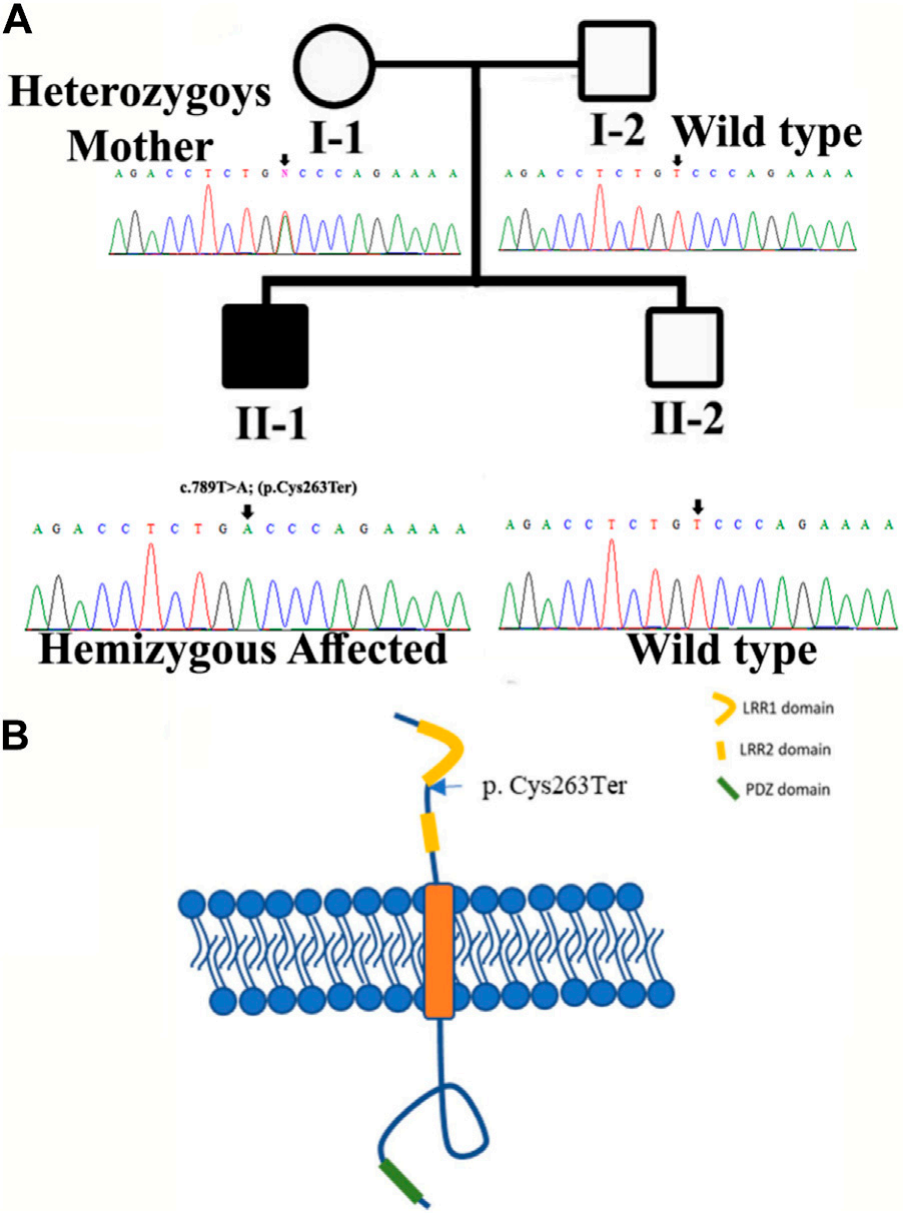
**(A)** Pedigree of the kindred segregating neurodevelopmental disorders. Circles represent females and squares represent male family members. The filled symbol designates the affected individual. Sanger sequencing results for each family member are also shown. **(B)** Structure of SLITRK2. The figure likely delineates different domains or regions within the *SLITRK2* gene. The labeled variant [p.(Cys263*)] specifies an alteration at position 263 of the SLITRK2 protein. “p.Cys263”denotes the affected amino acid sequence, and the asterisk indicates a premature stop codon, suggesting an early termination of protein synthesis due to the variant.

The present family, with a single affected individual (II-1) presenting neurodevelopmental disorders, was recruited from a remote region of the Balochistan province of Pakistan (Figure 1A). The studies involving human participants were reviewed and approved by KAIMRC. Written informed consent to participate in this study was provided by the patient/participants or the patients’/participants’ legal guardians/next of kin. Written informed consent was obtained from the individual(s) and/or minor(s)’ legal guardians/next of kin for the publication of any potentially identifiable images or data included in this article. Peripheral blood was collected in an EDTA Vacutainer from all the available individuals. Genomic DNA was extracted and quantified using standard methods (Irfanullah et al., 2015; Umair et al., 2016).

### Whole-exome sequencing

The Illumina HiSeq 2,500 platform was used to sequence the DNA of the affected member (IV-1). The human assembly hg38 (GRCh38) and Burrows–Wheeler Aligner (v 0.7.5) were used to align all the obtained reads. However, for variant calling, PINDEL and SAM tools with ExomeDepth were used. Subsequently, the final variant calling format file (VCF) was generated and uploaded to the Illumina base space online tool (basespace.illumina.com), which was used to analyze the data (Hayat et al., 2020; Umair et al., 2020).

### Sanger sequencing

The variant obtained through WES data analysis of all the available family members was sequenced using Sanger sequencing as described previously (Ullah et al., 2018; Ullah et al., 2019). Primers were designed using Primer3 version 0.4.0.

### Bioinformatics analysis

The allelic frequencies of the shortlisted variants were analyzed using different online web tools, including gnomAD, All of US, and BRAVO. The pathogenicity of the identified variants was checked using different online tools. The conservation of the variants across different species was checked using the NCBI-HOMOLOGENE database (https://www.ncbinlm.nih.gov/homologene).

### Protein structure prediction

SLIT and NTRK-like protein 2 protein modeling was performed using methods described previously (Ahmad et al., 2018). The amino acid sequence of SLIT and NTRK-like protein 2 (SLITRK2) was retrieved from the UniProt Knowledgebase database with accession number Q9H156 in FASTA format. AlphaFold (AF- Q9H156-F1, AlphaFold Protein Structure Database [ebi.ac.uk]) was used to predict the three-dimensional structure of the protein based on its amino acid sequence. The three-dimensional structure of the mutated protein was generated using the User Template option offered by SWISS-MODEL (Khan et al., 2022; Mahmood et al., 2023). The protein interactions of SLITRK2 with other proteins were accessed and studied using GeneMANIA, an online tool providing insights into the network and functional associations among proteins (Franz et al., 2018).

### RT-qPCR

To functionally validate the variant, total RNA was extracted from all the available family members to investigate relative SLITRK2 mRNA expression using GAPDH (DQ403057) as the internal control ‘housekeeping’ gene. cDNA was synthesized from total RNA using standard methods and a standard cDNA reverse transcription kit (Shah et al., 2020). The PrimerBank database (https://pga.mgh.harvard.edu/Parabiosys/) was used to design the primer pair (Forward, 5ʹ-GTA​TCT​CCA​GGC​CGA​CTA​CA-3ʹ; Reverse, 5ʹ-GAC​AAA​GCG​GAA​CAC​ATT​GC-3ʹ). PCR SYBRGreen Master Mix was used for the qPCR reaction, which was carried out using a Quant-Studio 6 Flex Real-Time PCR System. All the reactions were repeated independently and performed in triplicate, and data were analyzed using ExpressionSuite software version 1. GAPDH was used as an endogenous control.

## Results

### Clinical features

Index II-1 is a 9-year-old boy with no familial history of neurodevelopmental disorders. He was born at full term via C-section to a 23-year-old mother. The Apgar score was 7 at both 1 and 5 min. He had pneumothorax shortly after birth due to respiratory issues; however, he was discharged on the fifth day. According to his mother, the index had feeding difficulties that caused coughing and mild choking. He occasionally suffered from stomach pain.

His gross motor milestones and fine motor skills were delayed, and he is currently receiving therapy. His language development was delayed and he had pragmatic speech issues.

Additionally, he had mild intellectual disabilities, with IQ scores of 55–60, and faced challenges in adaptive functioning across multiple domains, including communication, self-care, social skills, home living, community use, self-direction, health, and safety. Furthermore, he had substantial neuropsychological issues, including ADHD, obsessive-compulsive behaviors, tantrums, anxiety, and autism (repetitive behaviors, insignificant socialization, and sensitivity to loud sounds). His anxiety has improved with low-dose fluoxetine. At birth, the patient parameters were as follows: weight, 2.85 kg (13th percentile [−1.14 SD]); length, 48.1 cm (22nd percentile [−0.77 SD]); and head circumference (HC), 2.82 cm (< first percentile [−14.5 SD]). Currently, at 11 years of age, his height is 143.6 cm (51st percentile [+0.01 SD]), his weight is 41.3 kg (70th percentile [+0.51 SD]), and his HC is 51.6 cm (<11th percentile [−1.20 SD]). Physical examination of the patient identified a prominent forehead, hypertelorism, and a high philtrum.

**FIGURE 2:**
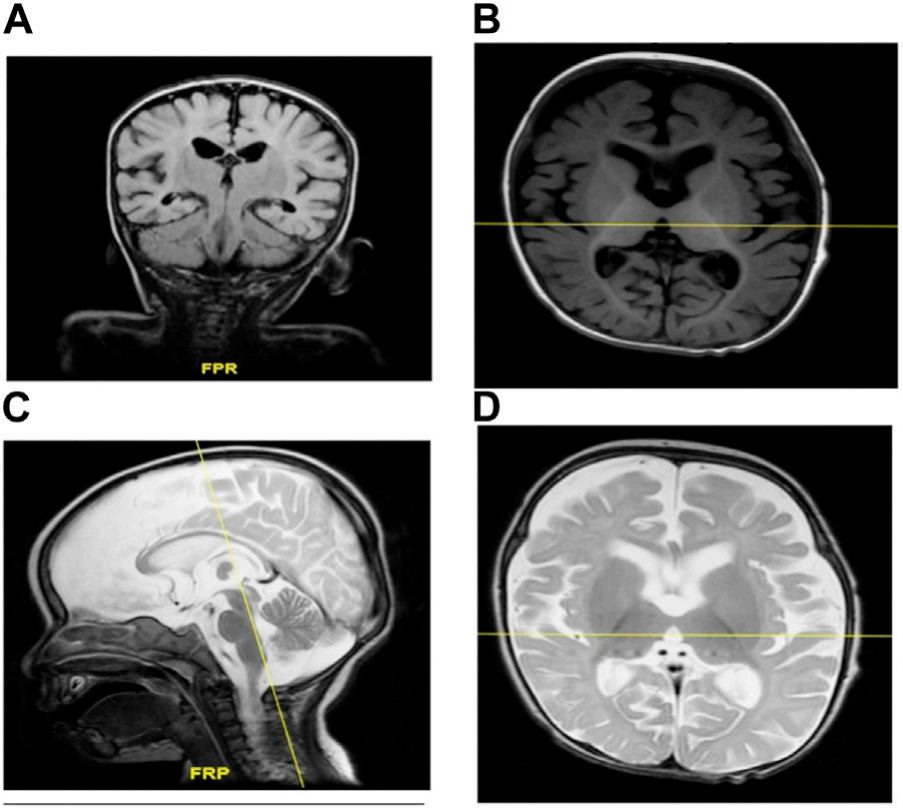
**(A–D)** MRI of individual II-1 revealed delayed white matter myelination, suggesting that the process of myelin formation in the white matter of the brain has not progressed at the expected rate for an individual of that person’s age. Additionally, the MRI revealed an underdeveloped corpus callosum. The corpus callosum is a vital structure that connects the left and right hemispheres of the brain, allowing for the communication and transfer of information between the two sides. An underdeveloped corpus callosum can lead to impaired communication and integration of information between the brain’s hemispheres, potentially affecting various cognitive functions, motor coordination, and sensory processing.

Brain MRI showed prominent CSF spaces and benign enlargement of the extra-axial CSF space *versus* brain parenchymal volume. Similarly, white matter myelination was delayed and the corpus callosum was also underdeveloped. Additionally, there was evidence of brain parenchymal volume loss (Figures 2A–D).

### Genetic analysis

**FIGURE 3:**
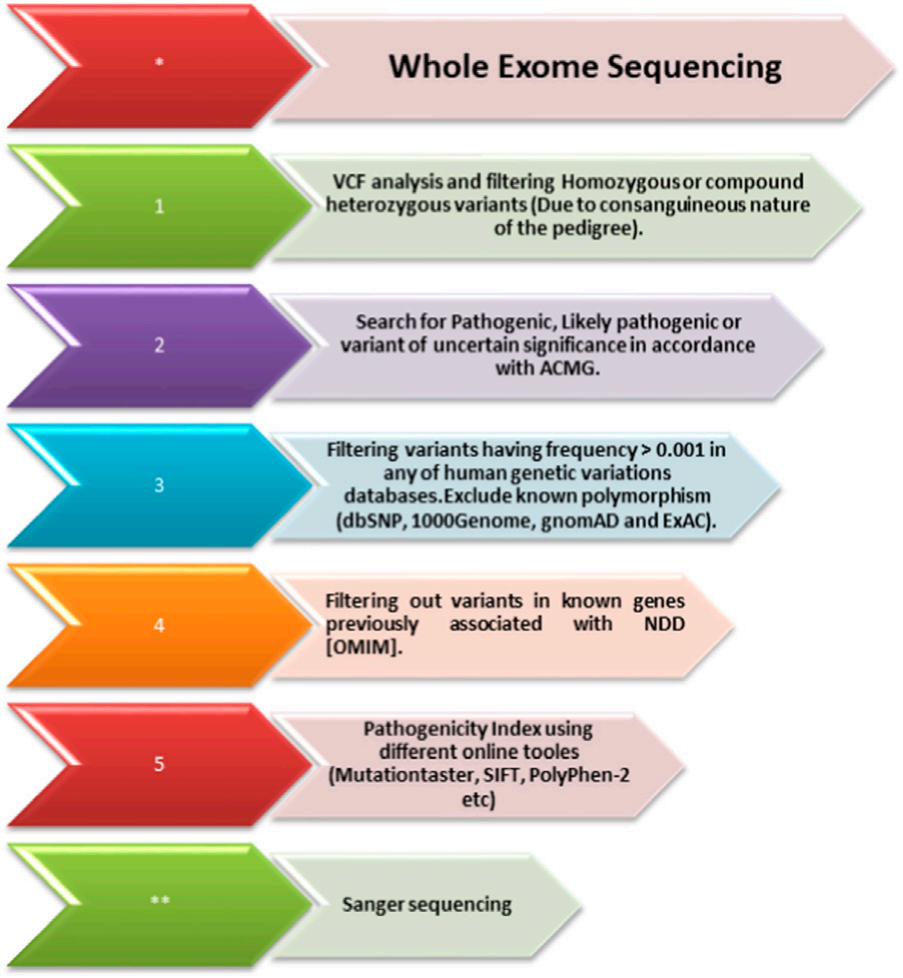
Whole-exome sequencing filtration steps. The steps, including analyzing the variant calling files to screen out homozygous and heterozygous variants, are shown. The variants are then classified according to the American College of Medical Genetics (ACMG) classification guidelines and further filtered using the minor allele frequency (MAF). After that, using USCS (known associated genes) and different online prediction tools, the pathogenicity of the variants was determined. Once the variant is screened through all these steps, Sanger sequencing of the variant in all the family members is performed using standard methods.

The DNA of an affected member (II-1; Figure 1A) of the family was subjected to exome sequencing. The variant filtration criteria were based on normal human database frequencies of ≤0.00, a CADD-phred score of ≥13, a splice site (+/-12bp), and variants located in the exons. A hemizygous nonsense variant [c.789T>A; (p. Cys263*)] within the *SLITRK2* gene was selected for further consideration due to its established association with intellectual disability. The variant was absent in normal human databases, including gnomAD v3.1.2, All of Us, and Bravo. In addition, it was absent in the variant database HGMD, which suggested that the variant was novel. The variant had a CADD phred score of 35 and GERP++ score of 5.22 and was predicted to be pathogenic by Mutation Taster and several other online tools. According to the ACMG, the identified variant was class 4 (likely pathogenic) (PVS1, PM2, PP1, and PP3) (Figure 3).

**FIGURE 4:**
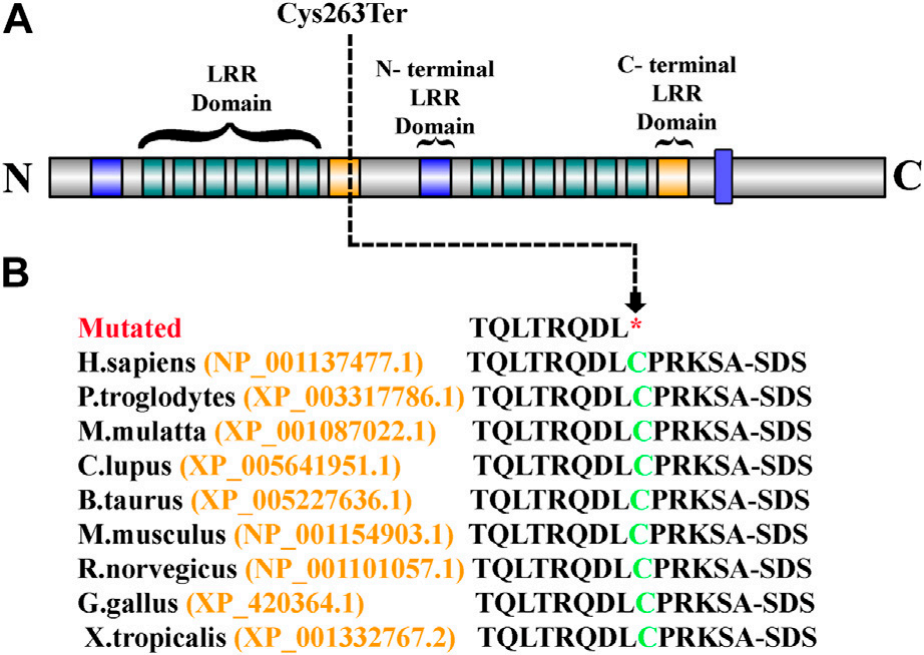
**(A)** Schematic representation of the SLITRK2 domains, which include the N-terminal LRR domain and the C-terminal LRR domain. The arrow shows the position of the identified variant (Cys263Ter) in the present study. **(B)** Partial amino acid sequence of SLITRK2. The green-colored Cys263 shows its conservation and important role across different species.

Sanger sequencing revealed that the variant perfectly segregated within the family. The affected child was hemizygous for the variant (II-1), the mother was healthy heterozygous (I-1), and both the father and sibling were wild type (I-2, II-2) (Figure 1A). The identified variant was located in the C-terminal LRR domain of the SLITRK2 protein (Figure 4A). Furthermore, Cys263 was conserved across multiple species (Figure 4B).

### Homology modeling

**FIGURE 5:**
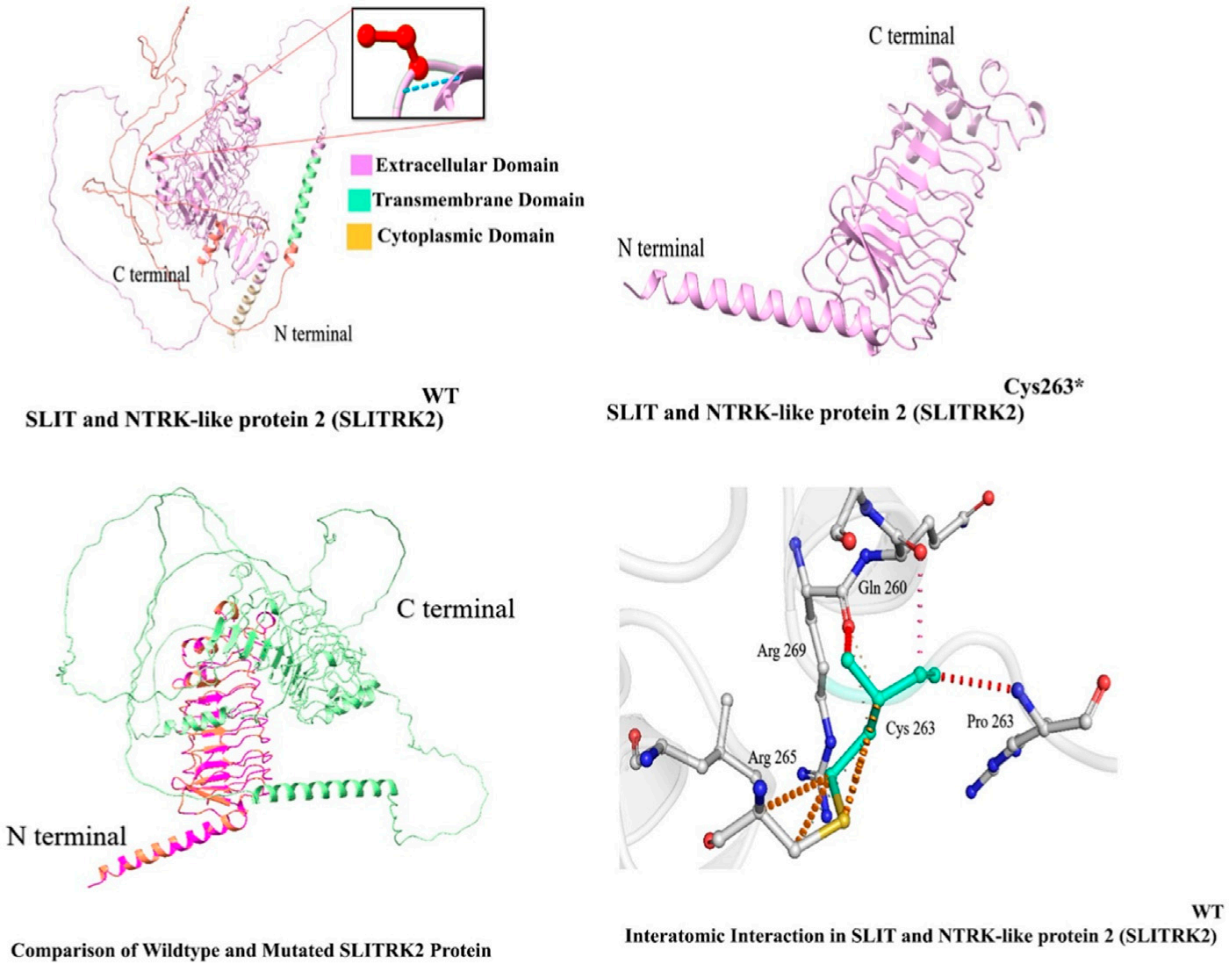
Predicted structure of SLITRK2 showing wild type (Cys263) and mutated protein (Cys263*). The three-dimensional protein structure of mutated SLITRK2 (Cys263*), suggesting key changes in the secondary structure that might result in a shorter and non-functional protein, which might result in degradation.

In this study, *in silico* methodologies, such as homology modeling for wild type and mutant, were carried out using standard methods. The predicted structure of SLITRK2 has a good degree of accuracy. Different evaluation programs assessed the final refined model. Using homology modeling, online structure analysis tools predicted and evaluated three-dimensional models of the wild type and mutated SLITRK2 [p.(Cys263*)]. Three-dimensional modeling revealed that the mutated SLITRK2 Cys263* truncated structure might not have proper function and perform downstream interactions (Figure 5A).

### SLITRK2 mRNA expression

**FIGURE 6:**
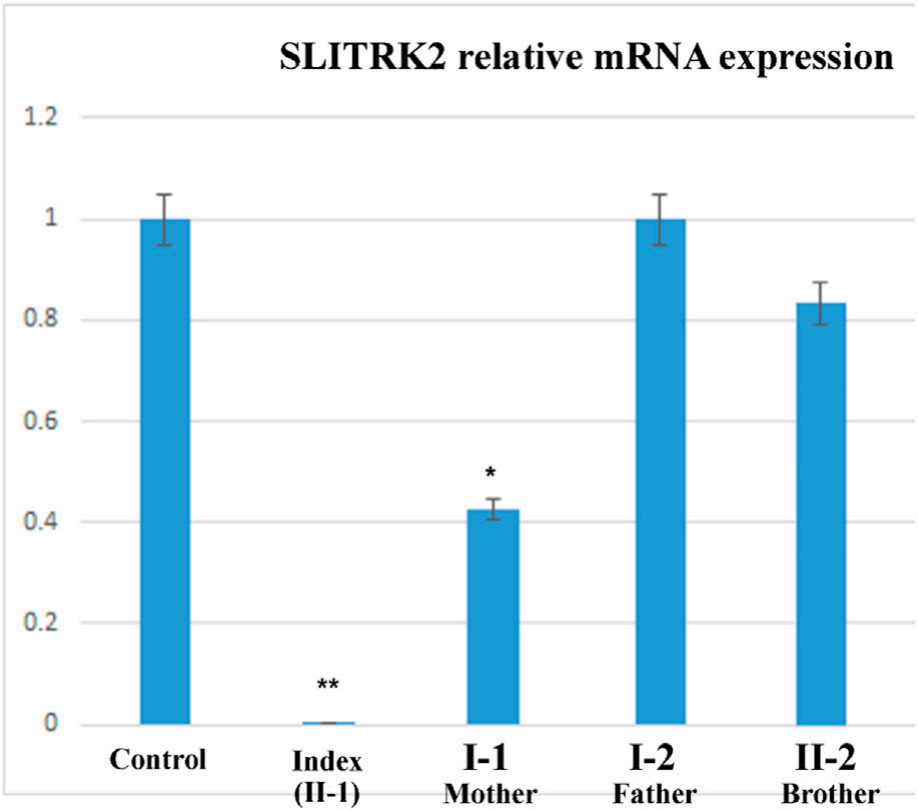
qPCR of the affected family showing a substantial decrease in the expression of SLITRK2 in the index compared with the other family members, showing that the variant identified in the index causes disease.

The relative expression data of the *SLITRK2* gene in the affected individual, parents, and normal control individuals showed that the proband (II-1) with the disease-causing variant [p.(Cys263*)] had substantially reduced *SLITRK2* gene expression compared with the wild type (control) and carrier (parents) (Figure 6).

### Discussion

The report, presented here, describes a family of Pakistani origin exhibiting hallmark features of neurodevelopmental disorders. Genetic analysis, including WES, revealed a novel nonsense variant [c.789T>A; (p. Cys263*)] in the *SLITRK2* (NM_032539.5) gene located on chromosome Xq27.3. Variants in the *SLITRK2* gene have been previously associated with X-linked intellectual developmental disorder type 111 (XLID111; OMIM 301107). Clinical phenotypes associated with XLID111 include short stature, microcephaly, feeding difficulties, kyphoscoliosis, developmental delay, speech delay, seizures, white matter anomalies, and ADHD (El Chehadeh et al., 2022).

SLITRK2, a single-pass transmembrane protein characterized by the presence of two leucine-rich repeat (LRR) domains within its extracellular domain and carboxy-terminal domains within its intracellular domain. Current variant [c.789T>A; (p. Cys263*); Figure 1B]; resides in the LRR1 domain of the protein and Cys263 is conserved among all SLITRK proteins. The LRR domains in the SLITRK family proteins are most like those of the Slit family, which are known to control axon branching and guidance (Brose and Tessier-Lavigne, 2000). Variants identified previously in the LRR domain of SLITRK2 have shown interrupting interactions of SLITRK2 LRR1 with LAR-RPTPs (Yamagata et al., 2015; El Chehadeh et al., 2022).

To date, a few variants have been identified in *SLITRK2*, which underlie neurodevelopmental disorders. In the current study, our patient manifested features as previously described, such as anxiety, ASD, and ADHD (El Chehadeh et al., 2022). In addition, the affected individual in the current study manifested facial anomalies, such as a prominent forehead, hypertelorism, and a high philtrum, that are yet to be associated with *SLITRK2* variants. Clinical variability reported in families might be due to epigenetics, genetic modifiers, and environmental factors (Khan et al., 2021).

**FIGURE 7:**
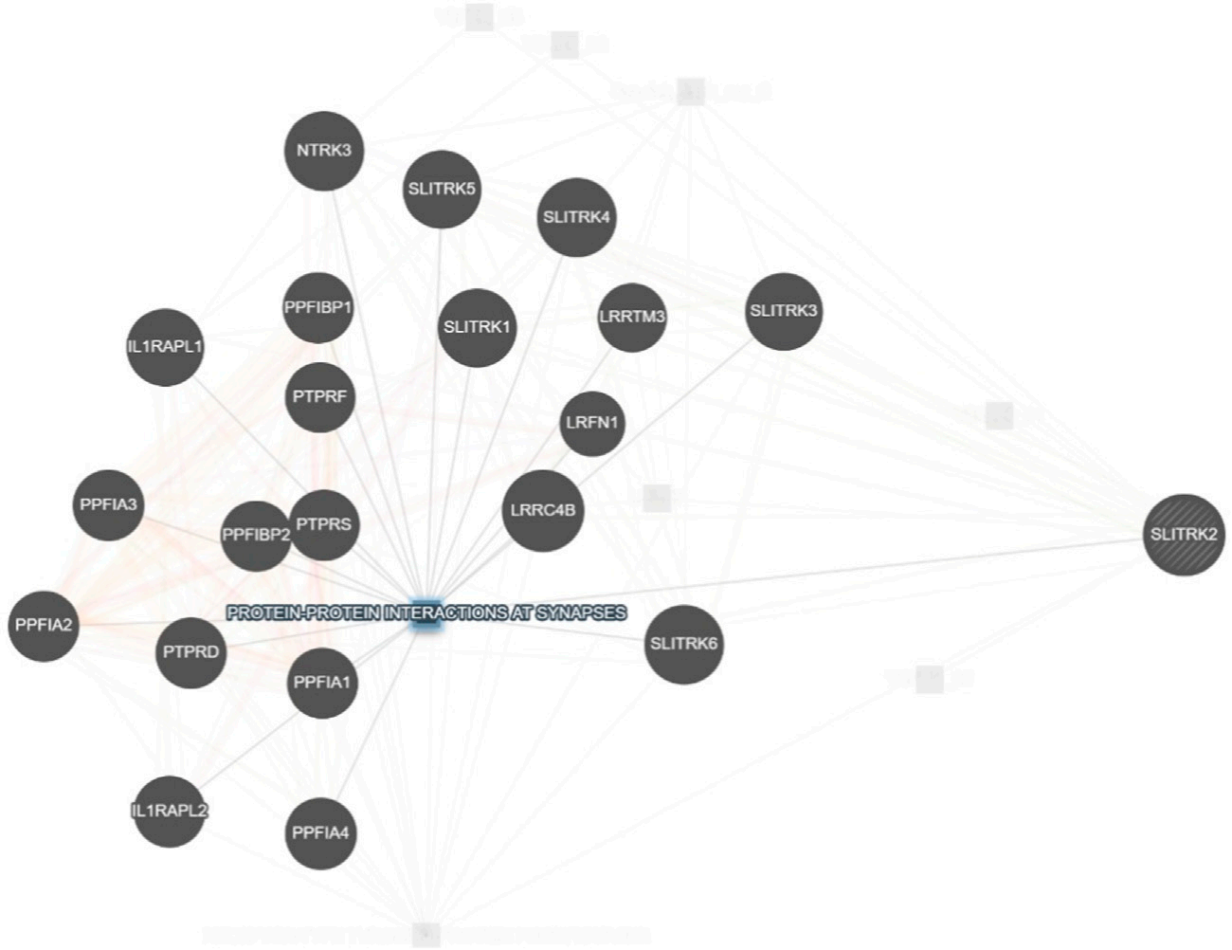
Interaction of SLITRK2 with other important players that might be associated with NDDs in our patient (https://genemania.org/), supporting the important and yet unexplored role of this gene in causing NDDs in humans.

SLITRK2 is a postsynaptic cell-adhesion molecule that promotes neurite outgrowth and excitatory synapse development (Han et al., 2019; Salesse et al., 2020). In terms of expression, SLITRK2 mRNA expression is highest in the pyriform cortex, olfactory regions, and hippocampus (especially the dentate gyrus). On a cellular level, SLITRK2 is highly expressed in mature cells of the CNS and immature neural progenitor cells (Aruga and Mikoshiba, 2003). The SLITRK2 protein was highly expressed in the hippocampus, vestibulocerebellum, and pre-cerebellar nuclei of the vestibular-cerebellar-brainstem neuronal network, which included the pontine gray and tegmental reticular nuclei. SLITRK2 knockout (KO) mice demonstrated higher locomotor activity in unfamiliar surroundings, antidepressant-like behaviors, improved vestibular function, and greater plasticity at mossy fiber-CA3 synapses with decreased serotonin sensitivity. Additionally, SLITRK2 KO mice exhibit antidepressant-like behavior, decreased long-term memory, and an aberrant gait. (El Chehadeh et al., 2022; Katayama et al., 2022). In humans, variants in SLITRK2 underlie skeletal anomalies, neurological abnormalities such as developmental delay, speech delay, and behavioral psychiatric symptoms (El Chehadeh et al., 2022; Table 1). Furthermore, SLITRK2 interacts with many important players involved in brain development in humans (Figure 7).

**TABLE 1:** Comparison of the clinical manifestations between current and previously identified variants in SLITRK2.

Details	El Chehadeh et al., 2022; P1	El Chehadeh et al., 2022: P2	El Chehadeh et al., 2022: P3	El Chehadeh et al., 2022: P4	El Chehadeh et al., 2022: P7	El Chehadeh et al., 2022: P8	El Chehadeh et al., 2022: P5	El Chehadeh et al., 2022: P10	Afsar et al. Present study
Sex	M	M	M	F	M	M	M	M	M
Mode of inheritance	*Denovo*	*Denovo*	X-linked	*Denovo*	X-linked	X-linked	X-linked	X-linked	X-linked
Ethnicity	French	French	Caucasian	Caucasian	Netherlands	Denmark	United States of America	United States of America	Pakistan
Age	30	28	21	13	12	11	12	08	09
Spastic	-	-	+	+	-	-	+	-	-
Dystonic	-	-	+	-	-	-	-	+	-
Ataxic	-	-	-	-	-	-	-	-	-
Hypotonic	-	-	-	-	-	-	-	-	-
Feeding difficulties	-	-	+	+	-	-	+	+	+
Kyphoscoliosis	-	-	+	+	+	-	+	-	-
Developmental delay	+	+	+	+	+	+	+	+	+
Speech impairment	+	+	+	+	+	+	+	+	+
ID	+	+	+	+	+	+	+	+	+
Seizure	-	-	+	+	-	+	-	-	+
Neuropsychiatric manifestations	+	+-	-	+	+	+	+	+	+
Facial abnormalities	-	-	-	-	-	-	-	-	+
Genomic position	c.1381G>T	c.1381G>T	c.934A>G	c.1276C>T	c.221T>C	c.1121C>G	c.1531G>A	c.628G>A	c.789T>A
Amino acid position	p.Glu461*	p.Glu461*	p.Thr312Ala	p.R426C	p.Leu74Ser	p.Prp374Arg	p.Val511Met	p.Glu210Lys	p.Cys263*
Transcript	NM_032539.4	NM_032539.4	NM_032539.4	NM_032539.4	NM_032539.4	NM_032539.4	NM_032539.4	NM_032539.4	NM_032539.4

In recent years, prenatal genetic screening and newborn screening received a boost due to affordable and rapid next-generation sequencing technologies (Alyafee et al., 2021a). Cell-free DNA (cf-DNA) sequencing using NGS technologies with the maternal plasma has led to the development of a highly sensitive screening test for fetal aneuploidies, and similarly, prenatal diagnosis for monogenic disorders (PGT-M) can be detected at an early gestation stage (Alyafee et al., 2021b; Alyafee et al., 2022). Thus, with the improvement in NGS-based sequencing technology, cost reduction, and data-analysis time reduction, cell-free nucleic acid sequencing would play an increasingly important role in the prenatal screening, diagnosis, monitoring, and risk stratification of fetal and maternal conditions (Umair, 2023). Furthermore, reporting new cases for the recently reported genes for neurodevelopmental disorders will not only add valuable data for genotype-phenotype correlation but also help clinicians to confidently screen patients and move forward with management and gene therapy (Nøstvik et al., 2021; Royer-Bertrand et al., 2021; Meng et al., 2023; Nishikawa et al., 2023).

In conclusion, we have reported a novel *de novo* variant in *SLITRK2* associated with neurodevelopmental disorders with additional features including facial abnormalities. This study will aid in expanding the variant spectrum of *SLITRK2*-related neurodevelopmental disorder and help with the proper genetic counselling of the affected family.

## Data Availability

The original contributions presented in the study are publicly available. This data can be found here: https://databases.lovd.nl/shared/variants/0000944763#00000888.
